# "Re-evaluation of variants of uncertain significance in patients with hereditary arrhythmogenic disorders"

**DOI:** 10.1186/s12872-024-04065-w

**Published:** 2024-07-27

**Authors:** Sarah Martin, Tina Jenewein, Christof Geisen, Stefanie Scheiper-Welling, Silke Kauferstein

**Affiliations:** 1 Centre for Sudden Cardiac Death and Familial Arrhythmias, Institute of Legal Medicine, University Hospital Frankfurt, Goethe-University, Frankfurt/Main, Germany; 2 DZHK (German Centre for Cardiovascular Research), Partner Site Rhein-Main, Frankfurt, Germany; 3 German Red Cross Blood Center, Institute of Transfusion Medicine and Immunohaematology, University Hospital Frankfurt, Frankfurt, Germany

**Keywords:** Arrhythmia syndromes, Variants of uncertain significance, Sudden cardiac death, Reclassification, Genetics

## Abstract

**Background:**

Genetic diagnostics support the diagnosis of hereditary arrhythmogenic diseases, but variants of uncertain significance (VUS) complicate matters, emphasising the need for regular reassessment. Our study aims to reanalyse rare variants in different genes in order to decrease VUS diagnoses and thus improve risk stratification and personalized treatment for patients with arrhythmogenic disorders.

**Methods:**

Genomic DNA was analysed using Sanger sequencing and next-generation sequencing (NGS). The Data was evaluated using various databases and in silico prediction tools and classified according to current ACMG standards by two independent experts.

**Results:**

We identified 53 VUS in 30 genes, of which 17 variants (32%) were reclassified. 13% each were downgraded to likely benign (LB) and benign (B) and 6% were upgraded to likely pathogenic (LP). Reclassifications mainly occurred among variants initially classified in 2017–2019, with rates ranging from 50 to 60%.

**Conclusion:**

The results support the assumption that regular reclassification of VUS is important, as it provides new insights for genetic diagnostics, that benefit patients and guide therapeutic approach.

**Supplementary Information:**

The online version contains supplementary material available at 10.1186/s12872-024-04065-w.

## Background

The worldwide incidence of sudden cardiac death (SCD) is 4–5 million cases per year [1] and accounts for 20% of all deaths in industrialized countries [2]. However, the cause of approximately 40% of all SCDs is unknown. Often, these deaths are due to hereditary arrhythmogenic diseases [3] and SCD is usually the first and only symptom.

The hereditary arrhythmogenic diseases are categorized into two main groups: one comprises primary structural diseases with an associated risk of arrhythmia, known as cardiomyopathies, while the other group consists of primary arrhythmia syndromes, often referred to as channelopathies. The cardiomyopathies comprise dilated cardiomyopathy (DCM), hypertrophic (obstructive) cardiomyopathy (HOCM, HCM), arrhythmogenic cardiomyopathy (ACM), arrhythmogenic right-ventricular cardiomyopathy/dysplasia (ARVC/D) and noncompaction cardiomyopathy (NCCM).

The field of channelopathies includes Long-QT syndrome (LQTS), Brugada Syndrome (BrS), early repolarization syndrome (ERS), catecholaminergic polymorphic ventricular tachycardia (CPVT) and short-QT syndrome (SQTS), [3–6].

Genetic diagnostics aim to identify these hereditary arrhythmogenic diseases and to treat them accordingly at an early stage. However, sequence variants are often discovered whose effect is still unknown. These variants are referred to as "variants of uncertain significance" (VUS). Such variants do not have sufficient evidence to confirm a clinical phenotype and no informed clinical decision can be made based on such a classification [7].

Re-evaluation of sequence variants, especially of VUS, should therefore be done regularly considering current data. Smith et al. reclassified three percent of all rare genetic variants after one year [8] and in a study by Campuzano et al., more than 70% of rare genetic variants were reclassified in 104 cases, from patients with arrhythmogenic diseases [9]. These studies highlight the importance of regular and continuous re-evaluation of rare sequence alterations for the diagnosis and treatment of patients with arrhythmogenic disorders. However, it is crucial that the studies were based on reclassifying a limited set of genes. Only a few studies have addressed these issues for a broad range of genes associated with inherited arrhythmogenic syndrome.

Since VUS are often observed with increased patient anxiety, inaccurate recollection of result due to a deficient understanding of the diagnosis, diminished rates of information sharing within the family and reduced uptake of family screening [7] continuous reclassification is recommended to update their role before clinical transition. In our study we focused on the reanalysis of these rare variants in a large set of genes and reclassification due to new available data. Our main objective is to reduce the frequency of VUS being identified as a diagnosis to the greatest extent possible. Therefore, the aim of our study is to improve risk stratification and personalized treatment of the patients through an extended reclassification based on current standard.

## Materials and methods

### Cohort

Our retrospective study reanalysed and reinterpreted 53 rare variants identified in 42 patients with suspicion of inherited cardiac diseases. These rare variants were all originally classified as VUS in our laboratory between 2016 and the beginning of 2022. All rare variants were identified in genes associated with arrhythmogenic diseases (BrS, LQTS, ARVC/D, DCM, DCM/NCCM, SCD, CPVT, ERS, ACM and HCM/HOCM).

The genetic analysis received approval from the ethics committee at the Department of Medicine, Goethe University. Clinical and genetic data concerning all patients were kept anonymous. Written informed consent was obtained from all patients including in the study before genetic analysis.

### Genetic analysis

Genomic DNA was extracted from EDTA blood samples using a salting out procedure. Concentration and purity were determined using the Nanodrop® ND-1000 Spectrophotometer v3.1.0 (Intas, Göttingen, Germany). From 2016 until the end of 2019, the exons and adjacent intronic regions (splice sites) were amplified by polymerase chain reaction (PCR) and directly sequenced by means of BigDye Terminator v1.1 chemistry and 3130xl Genetic Analyzer. (Applied Biosystems) Results were evaluated using SeqScape Software v2.5 (Life Technologies).

Since the end of 2019, Next-Generation Sequencing (NGS) was implemented using the TruSight cardio panel consisting of 174 genes with known cardiac associations as well as a custom enrichment-panel including nine genes by Illumina. Libraries were prepared using Nextera™ Flex Technology (Illumina). Sequencing was performed on the Illumina platforms MiniSeq or MiSeq system (2 × 150 bp paired end reads). Resulting reads were aligned to the GRChr37 (hg19) human reference genome. GensearchNGS software (Phenosystems®) was used for evaluation of the data.

Detected sequence variants were assessed using common databases: Exome variant server (EVS) [10], Genome Aggregation Database (gnomAD) [11] and applying in silico prediction tools (PolyPhen-2 [12], MutationTaster [13], SIFT [14], and CADD [15]). Variants in “core genes” (definitive association with disease) were confirmed by PCR and standard direct sequencing.

This renewal enables the sequencing of more genes in the individual panels. The genes analysed with the different techniques in the respective panels are listed in the additional file 1.

### Data

An exhaustive review of the literature and various databases concerning each variant was performed. Data were collected from HGMD [16], ClinVar [17] and National Center for Biotechnology Information SNP database [18]. All variants were consulted in varsome [19], Ensembl [20], Franklin [21], Cardio Classifier [22] and ClinGen [23].

### Classification

Variants were classified according to the standards of the American College of Medical Genetics and Genomics (ACMG) [24]. All variants were independently investigated by two genetic experts.

## Results

### Cohort

**Fig.1:**
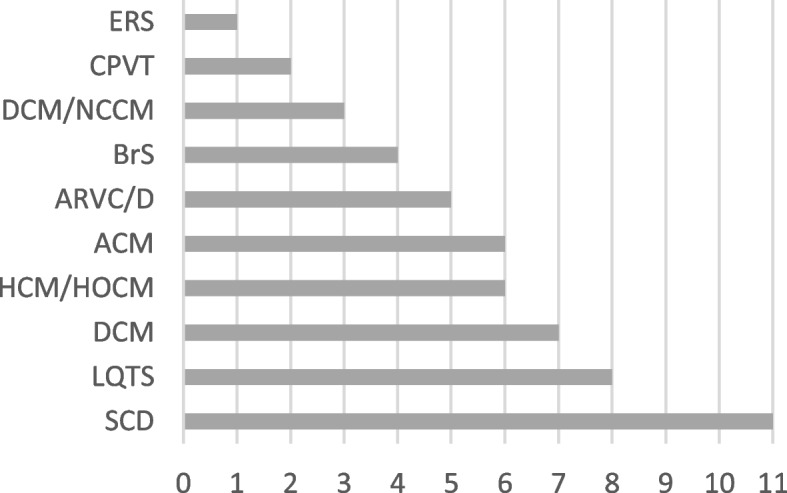
Distribution of VUS in the suspected primary indicated Diagnosis. VUS = Variants of unknown significance

In our retrospective analysis, we included 42 participants, aged between 2 and 85 years (mean age: 41.5 years). Among them, 26 were female (48%) and 28 were male (52%). The cohort includes 5 cases with a survived SCD or a family history of a SCD (12%) and following cases with suspected or confirmed diagnoses: 7 cases with LQTS (17%), 7 cases with DCM (17%), 6 cases with HCM/HOCM (14%), 4 cases with ACM (9%), 4 cases with ARVC/D (9%), 4 cases with BrS (9%), 2 cases with DCM/NCCM (5%), 2 case with CPVT (5%) and 1 case with ERS (2%) (Fig. 1).

### Genetics

The cohort comprises a total of 53 VUS (Supplementary Material 2). In the group of 42 patients, 31 individuals presented with a solitary VUS, 9 patients exhibited two VUS (cases 2, 14, 26, 27, 34, 35, 39, 41 and 42; Supplementary Material 2), and one patient demonstrated three VUS (case 10, Supplementary Material 2). The 53 VUS consist of 47 exonic variants and 6 intronic variants located within a splicing region. 46 of the exonic variants are missense and one generates a stop signal (case 39, p.(Glu300*)).

**Fig.2:**
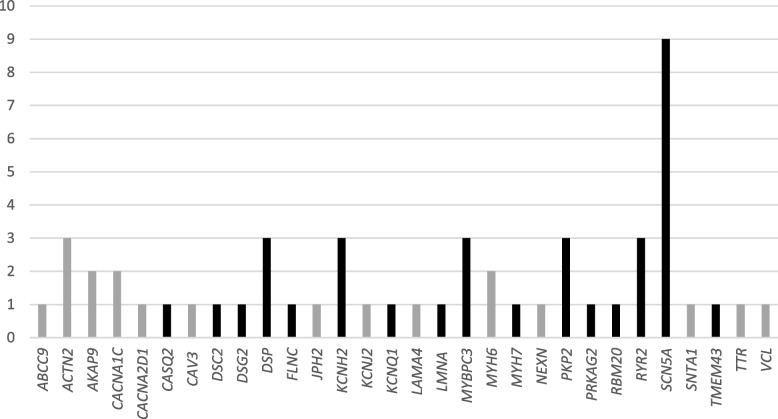
Number of VUS in the respective genes involved. Core Genes are highlighted in black. VUS = Variants of unknown significance

The 53 VUS were located in 30 genes (Fig. 2): 5 in *SCN5A*, 3 in *ACTN2*, 3 in *DSP*, 3 in *KCNH2*, 3 in *MYBPC3*, 3 in *PKP2*, 3 in *RYR2*, 2 in *AKAP9*, 2 in *CACNA1C*, 1 in *FLNC*, 2 in *MYH6*, 1 in *ABCC9*, 1 in *CACNA2D1*, 1 in *CASQ2*, 1 in *CAV3*, 1 in *DSC2*, 1 in *DSG2*, 1 in *JPH2*, 1 in *KCNJ2*, 1 in *KCNQ1*, 1 in *LAMA4*, 1 in *LMNA*, 1 in *MYH7*, 1 in *NEXN*, 1 in *PRKAG2*, 1 in *RBM20*, 1 in *SNTA1*, 1 in *TMEM43*, 1 in *TTR* and 1 in *VCL*.

Out of these 30 genes, 16 are referred to as core genes [23]: *CASQ2* in CPVT Autosomal Recessive (AR)) [25]; *DSC2*, *DSG2*, *DSP*, *PKP2* and *TMEM43* in ARVC [26]; *FLNC*, *LMNA* and *RBM20* in DCM [27]; *KCNH2* in SQTS and LQTS [27, 28]; *KCNQ1* in LQTS; *MYBPC3* and *PRKAG2* in HCM [29]; *MYH7* in HCM and DCM; *RYR2* in CPVT; and *SCN5A* in BrS, DCM and LQTS [30]. The core genes make up 53% of the genes involved in this cohort, with 59% of the reclassified variants found within these core genes (Fig. 2).

**Fig. 3:**
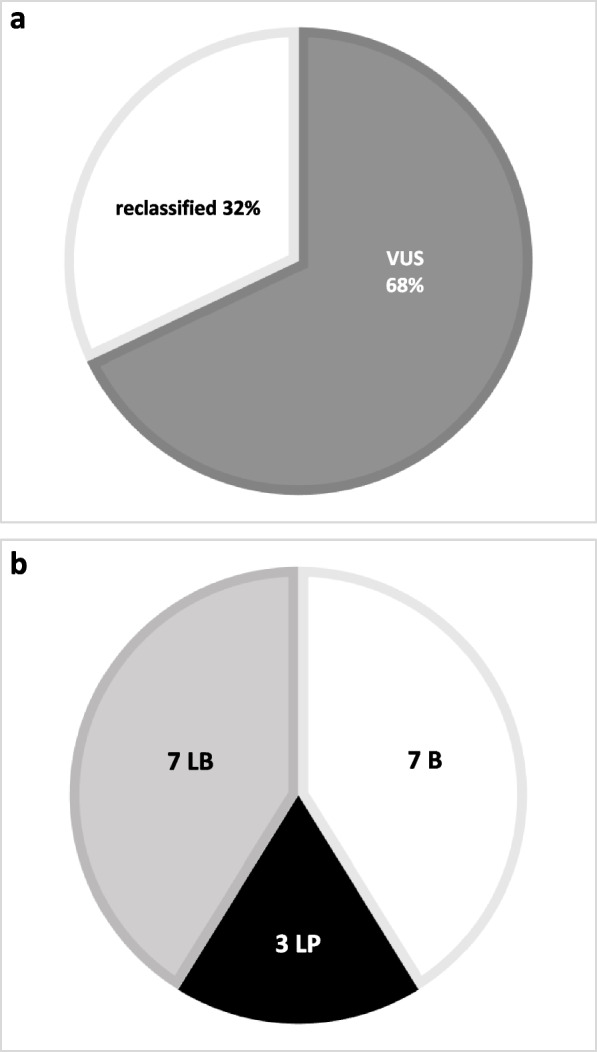
**a** Reclassification of VUS. White colour represents the percentage of variants that have been reclassified. Grey colour illustrates the percentage of variants that remained VUS. **b** Division of the VUS into LB, B and LP. White colour represents the variants that were reclassified as B, light grey colour represents the variants that were reclassified as LB and black colour represents the variants that were reclassified as LP. VUS = Variants of unknown significance, B = Benign, LB = likely Benign, LP = likely pathogenic

Reanalysis was performed by applying the ACMG guidelines and the usage of various databases and prediction programs (Supplementary Material 2). 17 sequence variants (32%) could be reclassified (Fig. 3a). 7 VUS (13%) each were downgraded to likely benign (LB) and benign (B) and 3 VUS (6%) were upgraded to likely pathogenic (LP) (Fig. 3b).

**Fig. 4:**
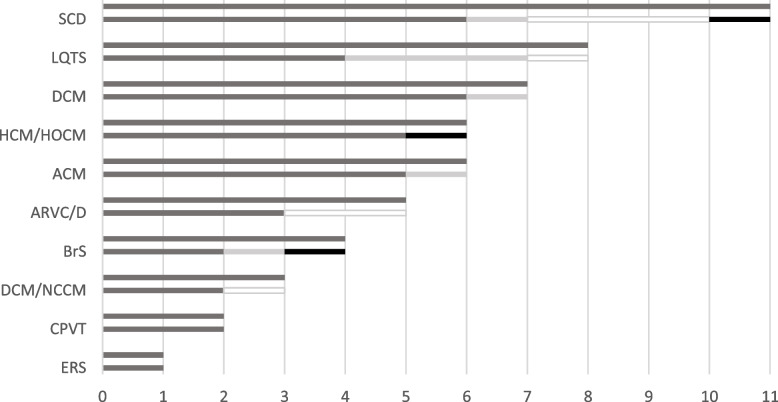
Reclassification in the respective gene panels. White colour represents the variants that were reclassified as B, light grey colour represents the variants that were reclassified as LB and black colour represents the variants that were reclassified as LP. Dark grey colour represents the VUS. VUS = Variants of unknown significance, B = Benign, LB = likely Benign, LP = likely pathogenic

There were 5 reclassifications in the gene panel for SCD, 4 in the gene panel for LQTS, 1 in the gene panel for DCM, BrS, and ARVC/D, 1 in the gene panel for HCM/HOCM, DCM/NCCM and ACM and 0 reclassifications in the gene panels for ERS and CPVT (Fig. 4).

The reclassification occurred especially among the VUS initially classified in 2017, 2018 and 2019. Out of the five VUS in our 2017 cohort, three were successfully reclassified (60%). Reclassification rates for variants from 2018 and 2019 were 50%. For the 2020 cohort 12.5% were reclassified, followed by 15% in 2021 and two out of two variants in 2022.

## Discussion

The prevalence of VUS in genetic testing results has prompted a need for comprehensive evaluation and understanding. Our analysis delves into several aspects that contribute to the complexity of interpreting VUS, providing insights into the relevance of a reliable revision of the interpretation of VUS in clinical genetic testing. In general, various aspects (for example functional studies, results of prediction tools and new databases entries) have been taken into account for the reclassification.

### Reclassification

Observing a substantial 32% of reclassification within our analysis of 53 VUS highlights the notable occurrence of periodic reclassification. In addition, our study contributes to the assessment that a considerable number of VUS detected in patients can be reclassified [31]. Recent studies have shown, however, that the majority of VUS are eventually reduced to benign or likely benign [7, 9, 32, 33]. While we can concur with our reanalysis that the trend of reclassification is leaning towards LB/B, it is important to note that a noteworthy of 6% of the variants have been reclassified as LP.

Those three variants were observed in patients with suspicion of BrS (case 5), HCM/HOCM (case 11) and SCD (case 39.1) (Supplementary Material 2).

In Case 5, the evidence of the variant p.(Ser106Gly) in the *SCN5A* gene pointed towards a potential causative impact on the protein. It was upgraded to LP including a functional study by Scheiper-Welling et al. This study supports the assumption that the variant affects channel function, potentially leading to life-threatening arrhythmias. The research highlights that the mutant variant induces certain abnormal functions, which may be directly associated with heart arrhythmias [34]. In summary, the study reinforces the detrimental effect on the gene or its product, thereby adding the ACMG-criterion PS3-supporting.

The variant p.(Arg27Pro) in the *CAV3* gene identified in case 11 was upgraded to LP based on a new submission to ClinVar (2022), which classified it as likely pathogenic. This led to the inclusion of the PP5 criterion outlined in the ACMG Guidelines, resulting in the reclassification from VUS to LP. As a result, the variant now includes the criteria PM2, PM5, PP3 and PP5.

The variant p.(?) in case 39.1 was initially already classified as potentially significant in 2021. Upon reviewing all prediction tools, which consistently indicated a likely pathogenic direction, we made the decision to upgrade the variant to LP.

These findings indicate that 18% of the reclassified variants now belong to the LP category. Hence, it is advisable to exercise caution in the clinical application of VUS, and it is recommended not to discard them, at least until further data are available to elucidate their potential clinical significance [9]. Given the potential impact of reclassification on the variant carrier, it is important that they receive updated diagnostic information [7] and, if necessary, for adjustments to be made in their therapy. For this purpose, an adapted report should be submitted to the sending physician in order to inform the patient of the latest findings, also with regard to possible further consequences. Additionally, genetic testing and clarification for other family members might be necessary.

Among the 54 VUS, seven were downgraded to B (cases 2.1, 2.2, 10.1, 10.2, 10.3, 26.1 and 42.1; Supplementary Material 2) and 7 were downgraded to LB (cases 6, 9, 14.2, 16, 21, 34.1 and 42.2; Supplementary Material 2). Out of all the downgrades to B, 43% had previously been categorized as potentially irrelevant (cases 10.2, c.80 C > T; 26, p.(Ser217Gly) and 42.1, p.(Phe2003Leu); Supplementary material 2). Similarly, 43% of the downgrades to LB had also been previously classified as probably benign (cases 16; 34.1, p.(Ile324Thr); and 42.2, p.(Phe2003Leu); Supplementary Material 2). The reclassification occurred after reviewing all prediction tools, which unanimously indicated the direction of the downgrade. Additionally, new entries were submitted to ClinVar in the majority of instances, either for a reclassification to B or LB. This introduces the criterion BP6 of the ACMG Guidelines into the assessment process and provides another basis for contemplating a downgrade.

Our results show that the reclassification of variants within our study occurred especially in the SCD panel, which encompasses genes associated with a wide range of heart conditions. The comprehensive search for variants within the SCD panel results in a significant proportion of identified VUS, accounting for 21% of the cases (Fig. 1). This leads to a wider inclusion of genes in the sequencing panels for SCD compared to more focused panels like ACM, which concentrate on genes directly linked to the condition, thereby resulting in a lower occurrence of VUS in these cases.

Our findings within the reclassification also endorse the periodic evaluation of VUS, aligning with other studies that suggest VUS should be reclassified at least every 5 years [7, 35]. While the precise timeframe for reclassification due to our small cohort and varied years of initial reclassification is challenging to determine, variants from 2019 and 2018 were reclassified by 50% and even 60% for those from 2017. Furthermore, a considerable number of VUS from the more recent years (19%), specifically 2020, 2021, and 2022, were also successfully reclassified. These outcomes support the notion that revaluation should occur at least every 5 years.

### *SCN5A* gene

An interesting observation arises when examining especially the *SCN5A* gene, as depicted in Fig. 2. Many of the involved VUS are located in the *SCN5A* gene. This gene has a high clinical validity, and it is included in several multi-gene panels. Thus, the *SCN5A* gene has been curated as definitive by the ClinGen expert panels for BrS, DCM and LQTS and classified as limited for ARVC. The main factor contributing to the high number of VUS in this gene is the comparatively higher background noise detected in healthy individuals. Thus, rare *SCN5A* nonsynonymous single-nucleotide variants (nsSNVs) among healthy individuals show a background rate of 2 to 5% [36, 37]. This compromises clinical genetic testing and therefore, determining the clinical relevance of these rare *SCN5A* variants becomes an even more difficult task [37].

Within our cohort, 9 VUS were identified in the *SCN5A* gene (cases 5, 8, 9, 26.2, 30, 31, 32, 41.2 and 42.2), accounting for 17% of the cases.

However, there are other factors that contribute to the elevated occurrence of the SCN5A gene and consequently, also to the presence of VUS. Firstly, variants in the *SCN5A* gene display a variable expression, resulting in a wide spectrum of clinical presentations including BrS, LQTS and DCM [38], while also exhibiting reduced penetrance. In our specific research, SCN5A was identified in individuals who underwent testing for BrS, DCM, SCD, LQTS, NCCM, and ACM (specifically, Supplementary Material 2 cases 5, 8, 9, 26.2, 30, 31, 32, 41.2 and 42.2). This leads to diverse phenotypic manifestations in a single gene [38, 39], complicating risk stratification and patient management [39]. This is further complicated by so-called overlap syndromes, in which a variant leading to a mixed phenotype that combines features of different cardiac diseases or genetic syndromes [39, 40]. Which means that individuals with identical *SCN5A* variants might manifest diverse phenotypes [41].This implies that various cardiac arrhythmias could potentially share a common origin [42]. Therefore, the classification of a variant often requires multiple gene carriers, as the same variant can lead to distinct phenotypes in different individuals. However, this is hindered in the case of many *SCN5A* variants due to their rarity and without additional evidence [43].

### Next-generation-sequencing versus sanger-sequencing

A general increased detection of VUS among patients can be explained, by the fact that there has been a rapid expansion of high-throughput genetic testing. While there is a chance to find more clinically significant variants, more VUS will be identified at the same time [36, 44].

In our analysis, 37 variants were assessed using NGS and 16 were subjected to Sanger sequencing. The benefits of NGS, including enhanced gene coverage and efficiency, have spurred its rising prominence in clinical diagnostics, evident in our study's transition from Sanger sequencing to NGS [29, 31, 45–52]. Thus, the number of genes was increased with the NGS examination method. As a result, the number of VUS has also increased due to the large scope of the examination. However, this fact is independent of the method and evaluation of the variant follows the same principle for both methods. It is important to emphasize, that the method itself had no impact on the results of our reclassification.

### Increased panel size

Current trends in expanding gene panels may lead to the identification of variants in genes with uncertain links to patient conditions. Precise classification of genes and variants is crucial to avoid potential genetic misdiagnoses and harm to patients [29, 30]. Careful gene selection, along with expert variant classification, is a crucial but often overlooked initial stage [24, 53–55]. Diagnostic gene panels should focus on definitively or strongly associated disease genes, with moderate associations requiring careful consideration. While genes with limited evidence may be useful in certain instances, their inclusion can contribute to uncertainty in clinical management and potential misapplications of genetic information [27, 29, 56–58]. Core genes, definitively associated with diseases, are vital for clinical genetic testing panels, as highlighted by the significant involvement of core genes in reclassifications [25]. Around 43% of the genes we analysed are core genes for the disease in question, and 59% of the reclassified variants are in these core genes. This underlines the importance of reclassifications, of which about two thirds concern syndromic core genes. Regular updates to gene panels are essential to reflect new insights and ensure therapeutic relevance, preventing the inclusion of ambiguous variants and reducing uncertainty.

### Effect of misinterpretation

In numerous instances, genetic testing serves as the pivotal factor in comprehending the underlying cause of disease and the results can impact the care of individuals at risk, potentially resulting in improved outcome as well as enabling the provision of suitable genetic counselling for family members [47, 59] and inaccurate interpretation of genetic data can lead to negative outcomes [55, 60]. Mistakenly diagnosing healthy individuals based on misinterpreted genetic results can result in unwarranted anxiety, unnecessary changes in lifestyle, adverse impacts on insurance coverage or job prospects, and, in certain situations, inappropriate medical interventions such as the placement of implantable cardioverter-defibrillators [28]. The primary challenge in interpreting VUS remains the subsequent clinical management of individuals [49, 61]. Additionally, the uncertainty surrounding the diagnosis of a VUS contributes to the difficulty in effectively conveying information about the genetic investigation to family members [31]. When diagnosing a VUS, there also exists a significant risk of either over-interpretation or under-interpretation [50] as well as the risk of the potential for them to become lost, meaning they might not be revisited and reclassified, even though this could be quite feasible a few years later, providing patients with a more precise understanding of their genetic condition [50]. The incomplete information accompanying VUS classification allows as well for a certain level of ambiguity in interpretation, thus warranting the consideration of the variant as not immediately actionable [7]. In principle, a VUS cannot be indicated as clinically relevant and therefore not as the genetic cause. In the context of research, family screening may be indicated, or if the core gene in which the VUS was found could match the clinically confirmed phenotype, the treating physician could recommend genetic testing to other clinically affected relatives. However, this assessment is a very sensitive undertaking and should be performed in expert centres.

## Conclusion

Our collected and analysed Data demonstrates that regular reclassification of VUS is significant, as new insights in genetic diagnostics can frequently lead to variant reclassification. These new findings hold great importance for patients and their family members in terms of guiding therapeutic approaches as well as help combat the psychological burden of ambiguity. Information about a reclassification should always be communicated to the patient, including any potential consequences. However, a structured re-calling programme is often not established. Considering the substantial changes observed in our study, revaluation should take place at least every 5 years.

## Limitations

Our study exhibits some limitations. Firstly, variant reclassification is subjective, as the interpretation of various VUS can vary among different laboratories. Secondly, this study overlooks the clinical findings, which could also hold significant implications for reinterpretation and reclassification. Furthermore, our cohort size is relatively small, and our findings should be validated through larger cohorts. Because of the small cohort and different years of the first diagnosis of the VUS it is not possible to identify the perfect time period between reclassifications. Additionally, we have not considered the time and costs associated with the regular reanalysis and reclassification of genetic variants. Also, the genes analysed vary between the two test methods. The number of genes has been adjusted over time in line with expert recommendation. The number of genes was increased with the NGS examination method. As a result, the number of VUS has also increased due to the larger scope of examination. However, this fact is independent of the method.

## Supplementary Information

Supplementary Material 1.

Supplementary Material 2.

## Data Availability

The data analysed as part of this study are contained in this published article and its supplementary information files. The raw data are available on request from the corresponding author. The human reference genome GRCh37/hg19 was used as the reference dataset in this study.

## Abbreviations

ACM: Arrhythmogenic cardiomyopathy
ARVC: Right-ventricular cardiomyopathy
ARVD: Right-ventricular dysplasia
BrS: Brugada syndrome
CPVT: Catecholaminergic polymorphous ventricular tachycardia
DCM: Dilated cardiomyopathy
ERS: Early repolarization syndrome
HCM: Hypertrophic cardiomyopathy
HOCM: Hypertrophic obstructive cardiomyopathy
LQTS: Long-QT syndrome
NCCM: Noncompaction cardiomyopathy
NGS: Next-generation sequencing
PCR: Polymerase chain reaction
SCD: Sudden cardiac death
VUS: Variant of uncertain significance
