# Correction: “Re-evaluation of variants of uncertain significance in patients with hereditary arrhythmogenic disorders”

**DOI:** 10.1186/s12872-024-04286-z

**Published:** 2024-11-01

**Authors:** Sarah Martin, Tina Jenewein, Christof Geisen, Stefanie Scheiper-Welling, Silke Kauferstein

**Affiliations:** 1 Centre for Sudden Cardiac Death and Familial Arrhythmias, Institute of Legal Medicine, University Hospital Frankfurt, Goethe-University, Frankfurt/Main, Germany; 2 German Red Cross Blood Center, Institute of Transfusion Medicine and Immunohaematology, University Hospital Frankfurt, Frankfurt, Germany; 3 DZHK (German Centre for Cardiovascular Research), Partner Site Rhein-Main, Frankfurt, Germany

**BMC Cardiovascular Disorders (2024) 24:390**

10.1186/s12872-024-04065-w

Following publication of the original article [1], the wrong Supplementary file “Supplementary Material 2” was originally published with this article; it has now been replaced with the correct file.

The original article has been corrected.

## Electronic supplementary material

Below is the link to the electronic supplementary material.

Supplementary Material 2
