# Supplementary material for: "Re-evaluation of variants of uncertain significance in patients with hereditary arrhythmogenic disorders"
Source: BMC Cardiovasc Disord. 2024 Jul 27;24:390. doi: 10.1186/s12872-024-04065-w (PMC11282671; doi:10.1186/s12872-024-04065-w)
Supplement: Supplementary file 1 — Supplementary Material 1. [file 12872_2024_4065_MOESM1_ESM.docx]

| Panels | PCR | NGS |
| --- | --- | --- |
| DCM | *MYH7, MYBPC3, TNNT2, TNNI3, LMNA, SCN5A, VCL* | *ACTN2, BAG3, FLNC, LMNA, MYBPC3, MYH7, RBM20, SCN5A, TNNT2, TNNI3, TPM1, TTN* |
| LQTS | *CACNA1C, CALM1, CALM2, CALM3, CAV3, KCNE1, KCNE2, KCNE3, KCNH2, KCNJ2, KCNQ1, SCN4B, SCN5A, SNTA1, TRDN* | *CACNA1C, CALM1, CAV3, KCNE1, KCNE2, KCNE3, KCNH2, KCNJ2, KCNQ1, SCN4B, SCN5A, SNTA1, TRDN* |
| HCM/HOCM | *CAV3, MYH7, MYBPC3, TNNT2, TNNI3* | *ACTC1, ACTN2, ANKRD1, CAV3, CSRP3, JPH2, LMNA, MYBPC3, MYH7, MYL2, MYL3, PLN, PRKAG2, TCAP, TNNC1, TNNI3, TNNT2, TPM1, SCN5A* |
| ACM | *DSP, DSG2, PKP2, RYR2* | *DSG2, DSP, PKP2, DSC2, JUP, SCN5A, CACNA1C, CAV3, KCNE1, KCNE2, KCNE3, KCNH2, KCNJ2, KCNQ1, LMNA, MYBPC3, MYH7, PLN, RBM20, RYR2, SCN4B, SNTA1, TGFB3, TMEM43, TNNI3 und TNNT2, TTNC1* |
| ARVC/D | *DSC2, DSP, DSG2, PKP2, RYR2* | *DSC2, DSG2, DSP, PKP2, JUP, SCN5A* |
| BrS | *CACNA1C, CACNB2, DSP, DSG2, GPD1L, KCNE3, MYBPC3, PKP2, RYR2, SCN1B, SCN3B, SCN5A.* | *CACNA1C, CACNB2, GPD1L, HCN4, KCNE3, SCN1B, SCN3B, SCN5A, TRPM4* |
| DCM/NCCM |  | *ABCC9, ACTC1, ACTN2, AKAP9, ANK2, ANKRD1, BAG3, CACNA1C, CACNA2D1, CACNB2, CALM1, CALM2, CALR3, CASQ2, CAV3, CRYAB, CSRP3, DES, DMD, DSC2, DSG2, DSP, FKTN, FLNC, GPD1L, HCN4, ILK, JPH2, JUP, KCND3, KCNE1, KCNE2, KCNE3, KCNE5, KCNH2, KCNJ2, KCNJ5, KCNJ8, KCNQ1, LAMA4, LAMP2, LDB3, LMNA, MYBPC3, MYH6, MYH7, MYL2, MYL3, MYLK2, MYOZ2, MYPN, NEBL, NEXN, PKP2, PLN, PRDM16, PRKAG2, RAF1, RANGRF, RBM20, RYR2, SCN10A, SCN1B, SCN2B, SCN3B, SCN4B, SCN5A, SGCD, SNTA1, TAZ, TCAP, TECRL, TGFB3, TMEM43, TNNC1, TNNI3, TNNT2, TPM1, TRPM4, TTN, VCL* |
| SCD |  | *ABCC9, ACTC1, ACTN2, AKAP9, ANK2, ANKRD1, BAG3, CACNA1C, CACNA2D1, CACNB2, CALM1, CALR3, CASQ2, CAV3, CRYAB, CSRP3, CTF1, DES, DMD, DPP6, DSC2, DSG2, DSP, DTNA, EMD, EYA4, FHL2, FKTN, FXN, GATAD1, GLA, GPD1L, HCN4, ILK, JAG1, JPH2, JUP, KCND3, KCNE1, KCNE2, KCNE3, KCNH2, KCNJ2, KCNJ5, KCNJ8, KCNQ1, LAMA4, LAMP2, LDB3, LMNA, MIB1, MYBPC3, MYH6, MYH7, MYL2, MYL3, MYLK2, MYOZ2, MYPN, NEXN, NKX2-5, PDLIM3, PKP2, PLN, PRDM16, PRKAG2, PTPN11, RAF1, RANGRF, RBM20, RYR2, SCN1B, SCN3B, SCN4B, SCN5A, SGCD, SNTA1, TAZ, TBX5, TCAP, TGFB3, TMEM43, TMPO, TNNC1, TNNI3, TNNT2, TPM1, TRDN, TRPM4, TTN, TTR, TXRND2, VCL* |
| CPVT |  | *CALM1, CASQ2, KCNJ2, RYR2 and TRDN; and analysis for ERS included: ABCC9, CACNA1C, CACNA2D1, CACNB2, GPDL1, HCN4, KCND3, KCNE3, KCNH2, KCNJ8, RANGRF, SCN1B, SCN2B, SCN3B, SCN5A, TRPM4* |
